# Primary Group A Streptococcus Peritonitis in a Healthy Child: A Case Report

**DOI:** 10.5811/cpcem.54019

**Published:** 2026-08-31

**Authors:** Alex Drinnan, James Naprawa, Sunghoon Kim, Karim Mansour

**Affiliations:** University of California, San Francisco, Benioff Children’s Hospital Oakland, Department of Emergency Medicine, Oakland, California

**Keywords:** group A streptococcus, spontaneous bacterial peritonitis, pediatric peritonitis, appendicitis mimic, case report

## Abstract

**Introduction:**

Group A streptococcus (GAS, *Streptococcus pyogenes*) is commonly associated with pharyngitis and skin and soft tissue infections, but invasive infection can occur. Primary bacterial peritonitis due to GAS is rare, especially in healthy children without nephrotic syndrome or immunocompromise.

**Case Report:**

We present a 21-month-old previously healthy male with four days of fever, irritability, and refusal to walk. Examination revealed abdominal distention and peritonitis. Computed tomography showed ascites and a normal appendix. Diagnostic laparoscopy revealed copious purulent peritoneal fluid; both peritoneal fluid and blood cultures were positive for GAS. He was treated with ampicillin-sulbactam followed by oral amoxicillin-clavulanate with complete recovery.

**Conclusion:**

This case highlights a rare presentation of invasive GAS as primary peritonitis in a healthy child. Clinicians should consider this diagnosis when peritonitis is present but imaging does not confirm appendicitis. Early surgical evaluation and targeted antimicrobials are essential.

## INTRODUCTION

Group A streptococcus (GAS; *Streptococcus pyogenes*) is an aerobic gram-positive coccus responsible for a wide range of clinical diseases.^1^–^3^ Although commonly associated with pharyngitis and superficial skin and soft tissue infections, GAS can also cause serious invasive infections including bacteremia, pneumonia, osteomyelitis, septic arthritis, necrotizing fasciitis, and streptococcal toxic shock syndrome.^1^–^3^ Invasive GAS infection occurs most frequently in young children, particularly those < 2 years of age.^2^,^3^ Primary bacterial peritonitis is uncommon in children, accounting for only 1–3% of cases of acute abdomen.^5^ Primary bacterial peritonitis is typically diagnosed in children with underlying comorbidities such as nephrotic syndrome or chronic liver disease.^5^–^7^ In these populations, *S pneumoniae* and *Staphylococcus aureus* are the leading causes.^5^–^7^ In contrast, primary bacterial peritonitis due to GAS in healthy children without predisposing conditions is exceedingly rare, with few pediatric cases described.^9^,^10^ We report a case of primary GAS peritonitis in a previously healthy toddler to highlight diagnostic challenges and emphasize the importance of early surgical and antimicrobial management.

## CASE REPORT

A 21-month-old previously healthy male presented with four days of fever, irritability, decreased oral intake, and refusal to walk. On day 1 of illness, he presented to the emergency department (ED) with a peak temperature of 104 °F and generalized shaking suggestive of a febrile seizure. He improved with antipyretics. On physical exam at the initial ED visit, he was alert and playful, and parents reported he was back to baseline. The abdominal examination was benign with no focal tenderness; parents reported the child was moving normally at that time. Rapid test results for severe acute respiratory syndrome coronavirus 2, respiratory syncytial virus, and influenza were negative. The clinical impression was a febrile illness with transient rigors, and the patient was discharged home with return precautions.

Two days later, he was seen by his primary care pediatrician for persistent fever. Parents reported that he also did not want to walk. On exam, he was able to ambulate but had a wide-based gait and possible ataxia. Pediatric neurology was consulted by phone and cerebellitis was considered; clinicians elected observation with referral to neurology if symptoms persisted or worsened. At that visit, lab testing revealed the following: leukocytosis, white blood cell count, 16.4 × 10^9^ per liter (L) (reference range, 5–15 × 10^9^/L); elevated C-reactive protein, 103 mg/L (< 10 mg/L); hemoglobin, 9.8 g/dL (10.5–13.5 g/dL); sodium, 128 mEq/L (135–145 mEq/L), and albumin 2.7 g/dL (3.5–5.0 g/dL). Given persistent fever and abnormal labs, the patient was referred to the ED for further evaluation.

On return to the ED, the patient appeared ill and uncomfortable. He had multiple episodes of watery, non-bloody diarrhea, and his parents noted that he stated, “everything hurts” when they lifted him. Vital signs were as follows: tachycardia, heart rate, 167 beats per minute; respiratory rate, 23 breaths per minute; temperature, 37.3 °C, and peripheral oxygen saturation, 95% on room air. Abdominal exam was notable for distention, diffuse tenderness, and involuntary guarding; parents had not noticed distention until it was pointed out.

Repeat laboratory evaluation demonstrated persistent leukocytosis with a white blood cell count of 16.1 × 10^9^/L) and C-reactive protein level was 376 mg/L, representing a marked elevation. Serum sodium was 134 mEq/L; albumin, 3.4 g/dL; and lactate 1.0 mmol/L (0.5–2.0 mmol/L). Abdominal ultrasound showed a small to moderate volume of complex free fluid predominantly in the lower abdomen; the appendix was not visualized. Computed tomography of the abdomen and pelvis with contrast demonstrated moderate ascites with a normal-appearing appendix and no periappendiceal inflammation.

Given concern for peritonitis, empiric ceftriaxone and metronidazole were administered, and pediatric surgery was consulted. The pediatric surgery attending examined the patient and recommended urgent diagnostic laparoscopy. Approximately 100 mL of purulent fluid was evacuated upon entering the peritoneal cavity. The appendix was grossly unremarkable aside from subtle tip inflammation without perforation. An appendectomy was performed and purulent fluid was aspirated for culture. Pathology showed mild acute inflammation of the appendiceal tip without perforation. Gram stain demonstrated gram-positive cocci on the external surface of the appendix. Peritoneal fluid culture and blood culture both grew beta-hemolytic group A streptococcus. Empiric ceftriaxone and metronidazole were transitioned to ampicillin-sulbactam, and the patient improved rapidly. He completed a 10-day course of antibiotics (intravenous, followed by oral amoxicillin-clavulanate) and was discharged on hospital day 5. At follow-up two days after discharge the patient was afebrile, playful, tolerating oral intake, and without abdominal symptoms.

*CPC-EM Capsule*

What do we already know about this clinical entity?

*Primary bacterial peritonitis is rare in healthy children and usually occurs in those with nephrotic syndrome or liver disease.*

What makes this presentation of disease reportable?

*Group A streptococcus caused primary peritonitis in a healthy toddler, mimicking appendicitis without perforation.*

What is the major learning point?

*Ascites with a normal appendix in a febrile child should prompt consideration of primary group A streptococcus peritonitis.*

How might this improve emergency medicine practice?

*Early recognition and surgical consultation allow targeted antibiotics and prevent delayed treatment.*

## DISCUSSION

This case represents an unusual instance of primary GAS peritonitis in a healthy child with no predisposing risk factors. Peritonitis in the pediatric population is most often secondary to appendicitis, intestinal perforation, or prior intra-abdominal surgery. When imaging demonstrates free fluid but no evidence of appendiceal rupture or other intra-abdominal source, the diagnosis becomes less straightforward. Primary peritonitis, defined as infection of the peritoneal cavity without an evident intra-abdominal source, is rare in children without nephrotic syndrome or liver disease.^5^–^7^

Appendicitis is the most frequent cause of pediatric peritonitis and is particularly challenging to diagnose in children < 5 years of age, in whom classic symptoms may be absent; up to 57% of cases in this group may present with perforation.^8^ Our patient’s imaging demonstrated ascites with a normal appendix, and surgical findings and pathology confirmed absence of perforation, making primary peritonitis the most likely diagnosis.

The pathogenesis of primary GAS peritonitis is not fully understood. Hematogenous spread from an unrecognized primary site, such as pharyngeal or cutaneous colonization, is the most accepted mechanism, with transient bacteremia after mucosal disruption allowing seeding of the peritoneum. Group A streptococcus virulence factors, including superantigens and exotoxins, likely contribute to invasive disease.^4^ In many reported cases, no clear antecedent focus is identified, as in our patient.

The existing literature includes only a small number of pediatric cases of primary GAS peritonitis.^9^,^10^ Most describe a sudden, severe presentation with abdominal pain, diffuse peritonitis, and, in some instances, hemodynamic instability requiring intensive care management.^9^ Dann et al reported two children presenting with abdominal pain and ascites in whom GAS was isolated from peritoneal fluid despite near-normal appendices.^9^ Sharp et al identified only 28 pediatric cases reported since 1975, underscoring the rarity of this diagnosis.^10^ Compared with prior reports, our patient had a more indolent course, suggesting that GAS peritonitis may present across a broader clinical spectrum than previously recognized.

Recognition of this entity is critical because GAS peritonitis may closely mimic perforated appendicitis. Clinicians should consider GAS when imaging demonstrates ascites without clear appendiceal inflammation, in the setting of systemic illness (eg, high fever and markedly elevated inflammatory markers). While empiric therapy for suspected perforated appendicitis often provides broad coverage, definitive therapy for GAS relies on penicillin-class agents once the organism is identified. Early differentiation between perforated appendicitis and alternative causes of pediatric peritonitis is critical in emergency practice. Key distinguishing clinical and imaging features are summarized in the table.

The patient’s initial ED visit was notable for a benign abdominal examination and transient clinical improvement after antipyretics; at that time, discharge with clear return precautions was a reasonable course of action. Pediatric illness is dynamic, particularly in preverbal children, and early examinations may not reveal evolving intra-abdominal pathology. This case highlights the importance of reassessment and low threshold to re-evaluate and obtain further testing when parents report new or worsening signs such as reluctance to ambulate, persistent high fever, or poor oral intake.

Difficulty ambulating has a broad differential in young children; abdominal pain may be an overlooked cause. Abdominal exams in preverbal children are challenging because these patients often cannot cooperate; paradoxically, peritoneal irritation may sometimes make the exam easier because the child resists movement. In our case, reluctance to ambulate likely represented early peritoneal irritation that evolved over days.

Multidisciplinary care involving emergency medicine, pediatric surgery, pathology, and infectious diseases facilitated timely diagnosis and effective management. Early attending-to-attending discussion and prompt intraoperative sampling were key to obtaining the microbiologic diagnosis and narrowing antimicrobial therapy.

## LIMITATIONS

This case report has several limitations. First, while primary GAS peritonitis is rare, it has been previously reported; therefore, this case does not represent a novel disease entity or a new diagnostic technique. Second, this was a single retrospective case with limited generalizability. Third, although hematogenous spread is the most plausible mechanism, the exact source of bacteremia was not identified. Finally, reflection on the initial decision to discharge is retrospective and may not capture all clinical factors considered at the time.

## CONCLUSION

Primary group A streptococcal peritonitis is a rare diagnosis in otherwise healthy children, but it carries important implications for emergency and surgical management. When children present with fever and peritonitis and imaging demonstrates ascites without evidence of appendiceal perforation, clinicians should consider this entity. Prompt recognition, early surgical consultation, and targeted antimicrobial therapy result in favorable outcomes.

## Figures and Tables

**Table:** Distinguishing features of pediatric peritonitis.

Feature	Acute appendicitis (± perforation)	Primary GAS peritonitis	Other etiologies (SBP, pneumonia, UTI, viral illness)
Typical age	Any age (↑ perforation risk <5 yrs)	Young children (rare)	Variable
Fever	Low early; higher with perforated	High early	Variable
Pain pattern	RLQ → diffuse if ruptured	Diffuse	Variable
Refusal to walk	Possible, especially if perforated	May occur early	Uncommon
Imaging findings	Dilated appendix, periappendiceal fat stranding ± small fluid	Moderate/free ascites with normal appendix	Variable
Blood cultures	Rare	May be positive	Rare
Mechanism	Luminal obstruction → perforation	Hematogenous spread	Varies

*GAS*, group A streptococcal; *SBP*, spontaneous bacterial peritonitis; *UTI*, urinary tract infection; *RLQ*, right lower quadrant; *yrs*, years.
